# Cells Assemble Invadopodia-Like Structures and Invade into Matrigel in a Matrix Metalloprotease Dependent Manner in the Circular Invasion Assay

**DOI:** 10.1371/journal.pone.0030605

**Published:** 2012-02-08

**Authors:** Xinzi Yu, Laura M. Machesky

**Affiliations:** The Beatson Institute for Cancer Research, Glasgow University College of Medical Veterinary and Life Sciences, Glasgow, United Kingdom; King′s College London, United Kingdom

## Abstract

The ability of tumor cells to invade is one of the hallmarks of the metastatic phenotype. To elucidate the mechanisms by which tumor cells acquire an invasive phenotype, in vitro assays have been developed that mimic the process of cancer cell invasion through basement membrane or in the stroma. We have extended the characterization of the circular invasion assay and found that it provides a simple and amenable system to study cell invasion in matrix in an environment that closely mimics 3D invasion. Furthermore, it allows detailed microscopic analysis of both live and fixed cells during the invasion process. We find that cells invade in a protease dependent manner in this assay and that they assemble focal adhesions and invadopodia that resemble structures visualized in 3D embedded cells. We propose that this is a useful assay for routine and medium throughput analysis of invasion of cancer cells in vitro and the study of cells migrating in a 3D environment.

## Introduction

The most common method currently employed to investigate cell invasion potential is probably the commercial Boyden chamber, modified with a thin layer of Matrigel, through which cells must also crawl to reach the other side of the filter [1]. Another inverted invasion assay [2], which involves a thicker layer of Matrigel (sometimes mixed with other components such as collagen or fibronectin) as the extracellular matrix (ECM) barrier and tissue culture medium (with serum or growth factors) as a chemoattractant allows quantitative analysis [3], [4]. However, neither of these methods is optimal for imaging, since the cells are either associated with a filter or fully inside of a 3D gel.

Formation of invadopodia on a thin gelatin matrix overlaid on glass is particularly amenable to imaging and has allowed the characterization of the dynamics and protein composition of these invasive protrusions of the cytoskeleton [5], [6], [7], [8]. Invadopodia are defined as actin-rich structures that also contain Arp2/3 complex, N-WASP and cortactin (among other proteins) and which have matrix degrading capacity [5]. Small punctate structures resembling invadopodia have been imaged in some 3D invasion systems [9], [10], supporting the idea that the relatively large and stable structures seen on thin matrix also exist although possibly more dynamically in 3D. In some aspects, invadopodia formed on thin gelatin resemble frustrated invasion attempts, as the cells never can actually crawl into the spaces that they degrade (they hit into the glass after a short distance). In 3D matrix, many cell types that make invadopodia in 2D will dig tunnels through the matrix and invade into these as collective chains termed invasion tunnels or single-cell invasion tunnels SCITS (single-cell invasion tunnels) [11]. The cells then form chains through the tunnels and make contact with each other as they migrate. This phenomenon has been compared with true collective invasion as is seen in tumors in vivo, where leader cells and stromal cells can make pathways that are used by follower cells [12], [13].

Focal adhesions are distinct from invadopodia and serve as the mechanical linkages to the ECM as well as hubs to integrate and direct numerous signaling proteins at sites of integrin binding and clustering [14]. Small focal complexes form in lamellipodia and some of them mature and enlarge into focal adhesions [15]. Once in place, a focal adhesion (FA) remains stationary with respect to the ECM, and the cell uses this as an anchor on which it generate force against the ECM. In a 3D matrix, focal adhesions and complexes (FC) are smaller and more dynamic than on a glass surface and have not been readily distinguished from each other [16]. While there was some controversy surrounding focal adhesion complexes in 3D [17], it is clear that these structures do form [18].

A circular invasion assay (CIA) was previously described to allow higher throughput and easier visualization of invading cells [19]. We have adapted and further characterized this assay and we propose that it is useful for comparison of cell migration parameters with cell invasion and for visualizing cells whilst they interact with a 3D matrix but still remain close to a glass surface. Cells in this assay invade in a protease dependent manner and assume the elongated shape of cells in 3D matrix. They assemble small focal adhesions that resemble those seen in 3D matrix [18] and also invadopodia-like structures containing cortactin, N-WASP, actin and Arp2/3 complex that associate with matrix degradations.

## Materials and Methods

### Cell culture and transfection

Cell culture reagents were purchased from Invitrogen (Paisley, UK). MDA-MB-231 breast adenocarcinoma cells and CHL-1 melanoma cells were obtained from ATCC. HT1080 fibrosarcoma cells were gifts from B. Ozanne (The Beatson Institute, Glasgow, UK). These cells were routinely cultured in complete DMEM supplemented with 10% fetal bovine serum (FBS) and 2 mM L-glutamine at 37°C in a humidified incubator with 5% CO_2_. Transfection of DNA plasmids and siRNA into these cells was performed by using the Amaxa “Nucleofector” system (Solution V, Programme X-013) according to the manufacturer's instructions.

### Antibodies and reagents

Antibodies were routinely used at 1∶1000 for western blotting and 1∶200 for immunofluoresence. Polyclonal rabbit anti-N-WASP was obtained from Atlas (Sigma). Monoclonal mouse anti-cortactin (4F11), polyclonal rabbit anti-p34-Arc (ARPC2) and monoclonal mouse anti-MT1-MMP were obtained from Millipore (Watford UK). Polyclonal rabbit anti-phospho-paxillin is from Cell Signalling Technology. Monoclonal mouse anti-vinculin is from Sigma-Aldrich. Monoclonal mouse anti-GAPDH is from Ambion. Rhodamine phalloidin, DAPI nucleic acid stain, DQ collagen, anti-mouse IgG and anti-rabbit IgG AlexaFluor antibody were obtained from Invitrogen (Paisley, UK). Horseradish peroxidase-conjugated secondary antibodies were obtained from Jackson ImmnoResearch Laboratories (Suffolk, UK). BD Matrigel™ basement membrane matrix is supplied by BD Biosciences.

### Constructs and siRNAs

The Cherry-MT1-MMP was a generous gift from Dr. Philippe Chavrier. The mouse GFP-constructs is a gift from Dr. Michael Way. N-WASP non-targeting (NT) control siRNA, ON-TARGETplus SMARTpool siRNA targeting MT1-MMP were purchased from Dharmacon.

### Immunoblotting

For western blot analysis, cells were lysed in RIPA buffer (50 mM Tris-HCl, 150 mM NaCl, 1% NP-40 and 0.25% Na-deoxycholate) with protease inhibitor cocktail (Pierce). Lysate were separated by SDS-PAGE and transferred to PVDF membranes (Amersharm). Western blotting was performed with the ECL chemiluminescence detection kits (Pierce) with appropriate species-specific horseradish peroxidase-conjugated secondary antibodies. The images were recorded and processed using GeneSnap software and Bio-imaging system (Syngene). Western blots shown in figures are representative of typical knockdowns obtained on multiple occasions for each experiment shown.

### Immunofluorescence staining in CIA

Cells in CIA were fixed in 4% formaldehyde for 30 min followed by permeabilization in 0.1% Triton X-100 for 20 min and blocking in 1% BSA. Primary antibodies were used at a 1∶200 dilution in blocking buffer. After 3 hours of incubation at room temperature or 16 hours 4°C (depending on the antibody) cells were washed extensively in blocking buffer, then secondary antibody was added at 1∶400 dilution in blocking buffer (plus fluorescently labeled phalloidin and DAPI if required) for 1 hour at room temperature.

### Inverted invasion assay

Inverted invasion assays were performed as previously described [2]. Briefly, BD Matrigel Basement Membrane Matrix (BD BioScience, concentration approx. 9 mg/ml) was mixed 1∶1 with PBS was allowed to polymerize in transwell inserts (Corning) for at least 1 hour at 37°C. Inserts were then inverted, and 5×10^5^ cells were seeded directly onto the outside surface of the filter. Transwell inserts were finally placed in serum-free medium, and medium supplemented with 10% FBS and 25 ng/ml EGF was added on top of the Matrigel to make a chemotactic gradient. 72 to 96 hours after seeding, invading cells were stained with Calcein-AM (Invitrogen) for 1 hour. The cells that did not cross the transfilter were removed with tissue and the remaining cells were visualized by confocal microscopy. Serial optical sections were captured at 15 µm intervals. The fluorescence intensity for each section was measured using ImageJ plugin Area Calculator. Finally, the index of invasion was calculated as the fluorescence intensity of cells invaded above 30 µm against the total fluorescence intensity of all cells within the images of sections taken. At least three independent experiments in duplicate were performed for each sample. To analyze cell morphology in the Matrigel, samples were fixed in 4% formaldehyde for 30 min, washed and permeabilized with 0.1% Triton X-100 for 30 min. Samples were then washed and stained with rhodamine phalloidin and DAPI overnight at 4°C followed by washing with PBS three times.

### Immunofluorescence staining of cells embedded in 3D collagen I

5×10^5^ MDA-MB-231 cells were suspended in 300 µl of PureCol pepsinised collagen I (3.3 mg/ml) (Advanced BioMatrix) and placed in a well of 24-well plate and allow to gel in 37°C for 2 hours. 500 µl of growth medium was added in to the well after the collagen was set. Cells were allowed to invade in the gel for 24 hours before fixation with 4% formaldehyde for 1 hour. After fixation, the plug of collagen with cells inside were placed into a 4 ml tube and permeabilized with with 0.1% Triton X-100 for 1 hour. Samples were then washed extensively and labeled with rhodamine phalloidin (1∶50) and primary antibodies (1∶100) overnight at 4°C followed by washing with PBS five times. Appropriate secondary antibodies were applied and incubated overnight at 4°C followed by extensive washing the next day.

### Time-lapse microscopy with modified circular invasion assay

For the modified Circular Invasion Assay (CIA) method, a square space (0.80 cm^2^) devoid of cells was created by placing a biocompatible silicon self-stick cellular stopper (Thermoscience, Ibidi, 80209) in the center of a 35 mm glass bottom dish (Ibidi) before seeding 6×10^5^ MDA-MB-231 cells. After cells adhere, the stopper is removed and 250 µl of 50% BD Matrigel™ (4.5 mg/ml) in PBS was overlaid onto the cell monolayer seeded in the inner circle of the dish to create a matrix barrier (0.8 mm high) against the cellular surface and allowed to polymerize for 2 hours prior to adding growth medium on the top of the set Matrigel. Monolayers with overlaid Matrigel, were then imaged with a Nikon time-lapse microscope or incubated in a humidified atmosphere of 5% CO_2_ at 37°C for 24 hours prior to fixation and immunofluorescence. Cells were tracked using ImageJ plugin Manual Tracking and the tracking results were analyzed using ImageJ plugin Chemotaxis Tool to calculate cell speed and invaded area. This quantification was done in least three independent experiments for each assay.

## Results

### Cells assume an elongated shape and invade in the modified circular invasion assay

**Figure 1:**
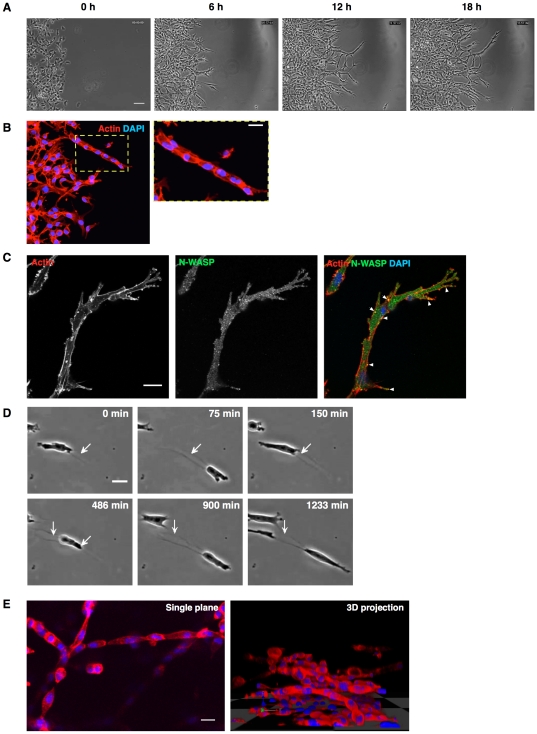
MDA-MB-231 cells form collectively invading chains in CIA. (A) Photos from a time-lapse video of MDA-MB-231 cells invading into Matrigel in CIA showing the formation of long invasion chains at the invading wound edge. Scale bar 50 µm. (B) Cells in a large finger-like chain at the leading edge of a CIA are shown fixed and stained with actin (red), and DNA (blue). Scale bar 20 µm. (C) Actin (red), N-WASP (green) and DAPI (blue) staining of a cell chain at the front of the invading area. White arrowheads indicate puncta of N-WASP co-localizing with filamentous actin. Scale bar 20 µm. (D) Image sequence showing an invading cell actively remodeling the matrix and generating what appear as micro-tunnels (white arrow). Scale bar 20 µm. (E) Staining of actin (red) and DNA (blue) of cell invasion chains in inverted invasion assay and visualized by confocal microscopy. Image showing collective cell invasion chains on single plane (left panel) and the side view of z-stack 3D projection (right panel). Scale bar 20 µm. See also Movies S1, S2, S3.

We have further developed and more extensively characterized the wound closure-based circular invasion assay as an effective method for visualizing cells during the invasion process [19]. As an alternative to wounding, we used a square, self-stick silicone cell stopper to create a uniform and neat central cell-free space without damaging or disrupting cells. MDA-MB-231 cells were seeded on a 35 µm glass-bottom dish with a stopper blocking the central area. After the cells attached to the glass bottom, the stopper was removed and a thin layer of Matrigel was applied on top of cells and allowed to set. Cells were generally invaded into the Matrigel for 16 hours or longer. As shown in Fig. 1A and B and Movie S1, MDA-MB-231 cells migrated into the central space, penetrating into the Matrigel while assuming an elongated shape near the invading front. Unlike traditional invasion assays, cells could be straightforwardly fixed and stained with antibodies to obtain high quality images. Fig. 1B shows phalloidin and DAPI labeling of leading edge invasion chains. Fig. 1C shows phalloidin stain of actin (red) and punctate appearance of endogenous N-WASP staining (green) with DAPI stain of DNA in blue. N-WASP and actin frequently co-localized to punctuate structures at the cell periphery and protrusions (Fig. 1C, white arrowheads). Invading cells formed cylinder-shape pseudopods and appeared to actively reshape the surrounding matrix by creation of tunnels resembling previously described micro-tunnels also termed SCITS (single-cell invasion tunnels) (Fig. 1D) [20]. Collective migration chains formed as following cells filled into the micro-tunnels (Fig. 1A–C, arrows indicate in C and Movie S1 and S2). These invasion chains resemble those that we and others have observed in 3D inverted invasion assays ([21] and Fig. 1E and Movie S3).

### Cells in CIA have small focal adhesion complexes and long thin pseudopodia, unlike in 2D on glass

**Figure 2:**
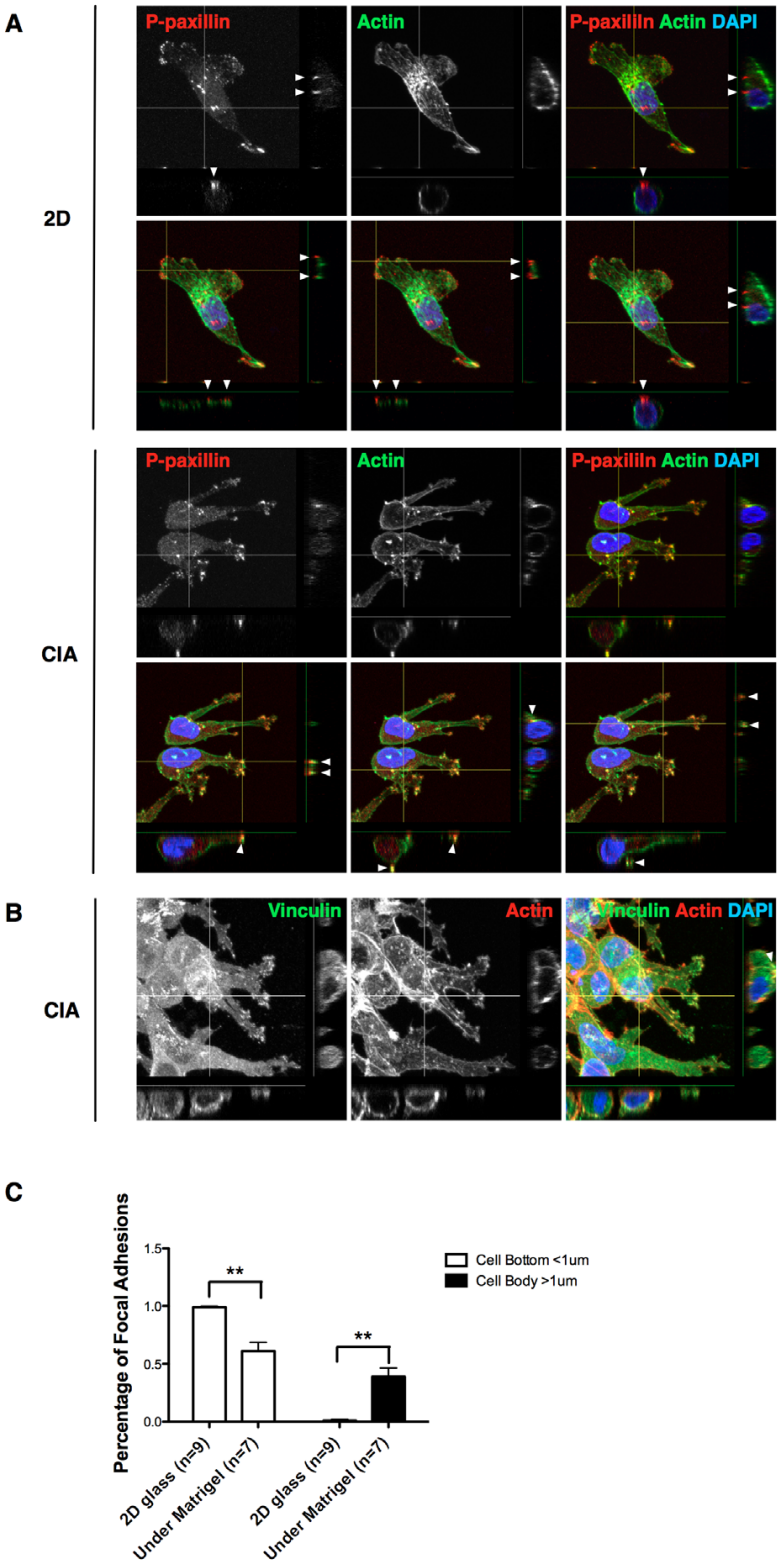
Actin cytoskeletal and focal adhesion organization in MDA-MB-231 cells invading in CIA. (A) Cells in wound healing assay without Matrigel on 2D surface and cells in CIA with Matrigel overlay were fixed and stained for actin (green), focal adhesion marker phospho-paxillin (red) and DNA (blue). Z-stack confocal images were captured and cell side views are shown to indicate positions of FA/FCs. White arrowheads indicate adhesion complexes. (B) Cells invading in CIA were fixed and stained with actin (red), focal adhesion marker vinculin (green) and DNA (blue). Z-stack confocal images were captured and cell side views are shown to indicate positions of FA/FCs. White arrowheads indicate adhesion complexes. (C) Quantification of adhesion complexes (puncta stained with phospho-paxillin) at the bottom of the cells (within 1 µm range above the glass) and on the cell body or on top of the cells (above 1 µm) under both conditions. All error bars indicate means ± SD; **, P<0.01 by Student's t-test. See also Movies S5 and S6.

We compared the morphology of MDA-MB-231 cells in the CIA with and without Matrigel overlay. Without matrix, cells are well spread and form fan-like lamellipodia actin protrusions, while the cells in CIA invading under Matrigel assume an elongated shape that resembles cells in a 3D matrix (Fig. 2A–C and Movie S2 and S4). We used FA/FC markers p-paxillin and vinculin to compare the distribution of adhesions in cells migrating on 2D surfaces with cells in CIA under Matrigel. When cells were migrating on glass, small FC puncta were present in lamellipodia and larger FAs were distributed on the basal side of the cell (Fig. 2A and Movie S5). In CIA with Matrigel, phospho-paxillin and vinculin clustered primarily in small puncta near the tips of invading pseudodpods and throughout the cell body (Fig. 2A–B and Movie S6). Interestingly, these phospho-paxillin clusters co-localized with filamentous actin puncta, unlike focal adhesions in 2D. Using Z-stack confocal microscopy, we investigated the positioning of these FA/FC with respect to the glass surface or in the case of CIA, the surrounding matrix. Of cells migrating on 2D rigid surface, almost 100% of the FAs were located on the basal surface of the cell within 1 µm of the substratum as shown in (Fig. 2A and C and Movie S5). While in cells invading in CIA, about 60% of FAs reside near the glass, but a considerable proportion of them (about 40%) reside above the bottom 1 µm of the cells. Many of them localize at the cell dorsal surface and along the invading pseudopods, suggesting close contacts with the surrounding ECM (Fig. 2B and C and Movie S6).

### Cell invasion in CIA requires matrix metalloprotease activity and MT1-MMP

**Figure 3:**
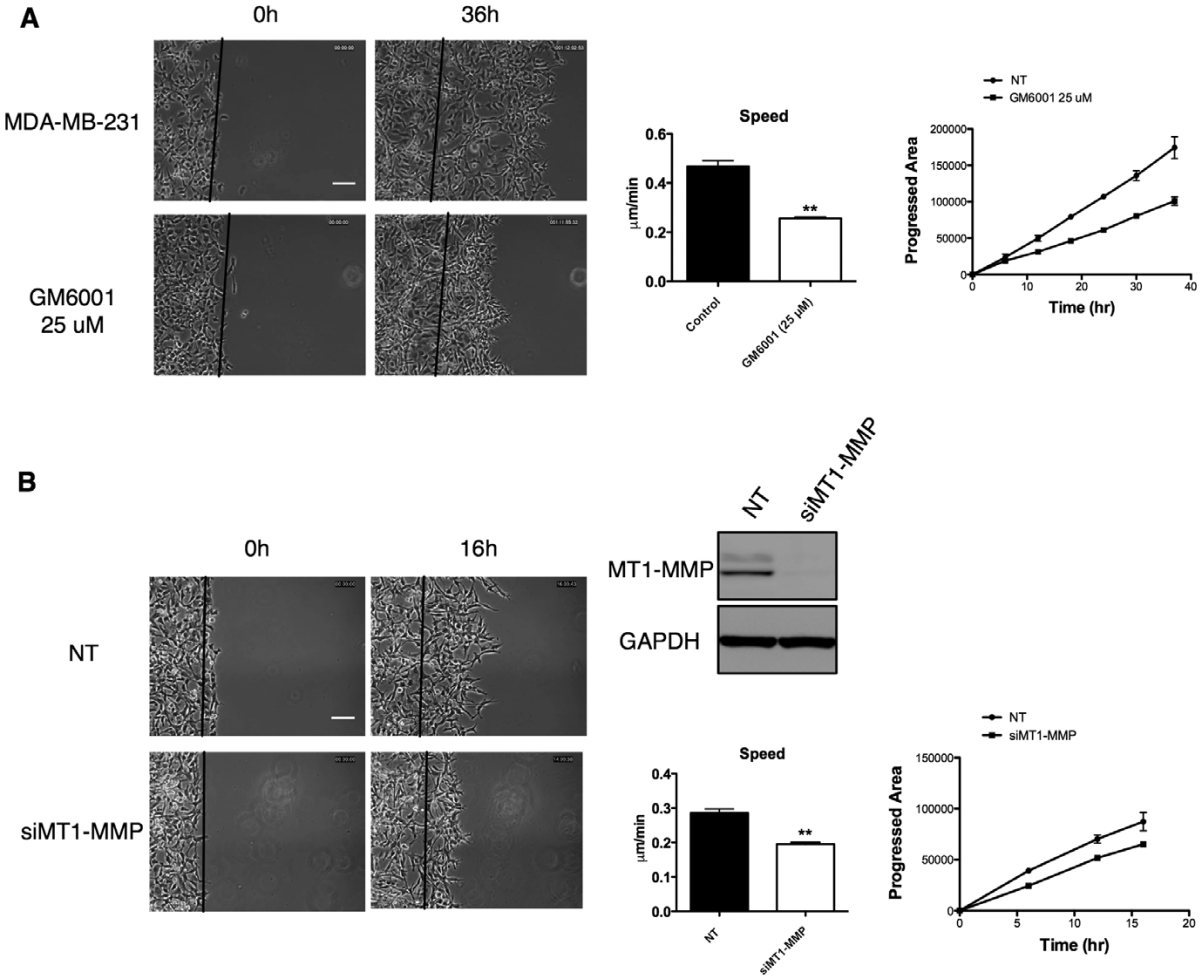
Cell invasion in CIA with Matrigel is an MMP dependent process. (A) Addition of 25 µM GM6001 significantly impairs MDA-MB-231 cell invasion in CIA speed and progressed area (arbitrary units) versus time are shown in the graphs. Scale bar 50 µm. (B) siRNA knockdown of MT1-MMP significantly impairs invasion in CIA. Western blot shows a representative knockdown of MT1-MMP. Movies were analyzed in three independent experiments. All error bars indicate means ± SD; **, P<0.01 by Student's t-test. Scale bar 50 µm. See also Movie S7.

To test whether migration in CIA is matrix metalloprotease (MMP) dependent, we monitored cell migration with addition of MMP broad-spectrum inhibitor GM6001. Normal medium in the CIA was replaced with medium containing 25 µM GM6001 and followed by time-lapse microscopy. As shown in Fig. 3A, GM6001 significantly retards migration of MDA-MB-231 cells into the central wound area resulting in reduced invading speed (Fig. 3A and Movie S7). Quantification of the cell speed and area covered by progressing cells revealed that cells treated with GM6001 invade into the wound area significantly more slowly than the control cells (Fig. 3A and Movie S7).

We also depleted MT1-MMP using siRNA and tested whether inhibition of major pericellular collagenase MT1-MMP alone is sufficient to impair cell ability to invade in CIA under Matrigel. Depletion of MT1-MMP also significantly (around 30%) impaired cell invasion in under Matrigel assay (Fig. 3B). Similarly, siRNA of MT1-MMP or addition of GM6001 to the medium significantly slowed down invasion of MDA-MB-231 cells into Matrigel in a 3D invasion assay [4]. Thus, MMP activity makes a significant contribution to cell invasion under Matrigel in this assay and in 3D assays using Matrigel.

### Cells assemble invadopodia-like puncta in CIA

On thin gelatin matrix, cells make invadopodia, actin-rich protrusions that degrade ECM [22], [23], [24], [25]. Invadopodia assembly is believed to be a default strategy for cancer cells to invade across basement membrane and through dense ECM. Because cell invasion in CIA requires MMP-mediated degradation, we investigated whether invadopodia structures may be involved in cell remodeling and degrading matrix in this assay.

**Figure 4:**
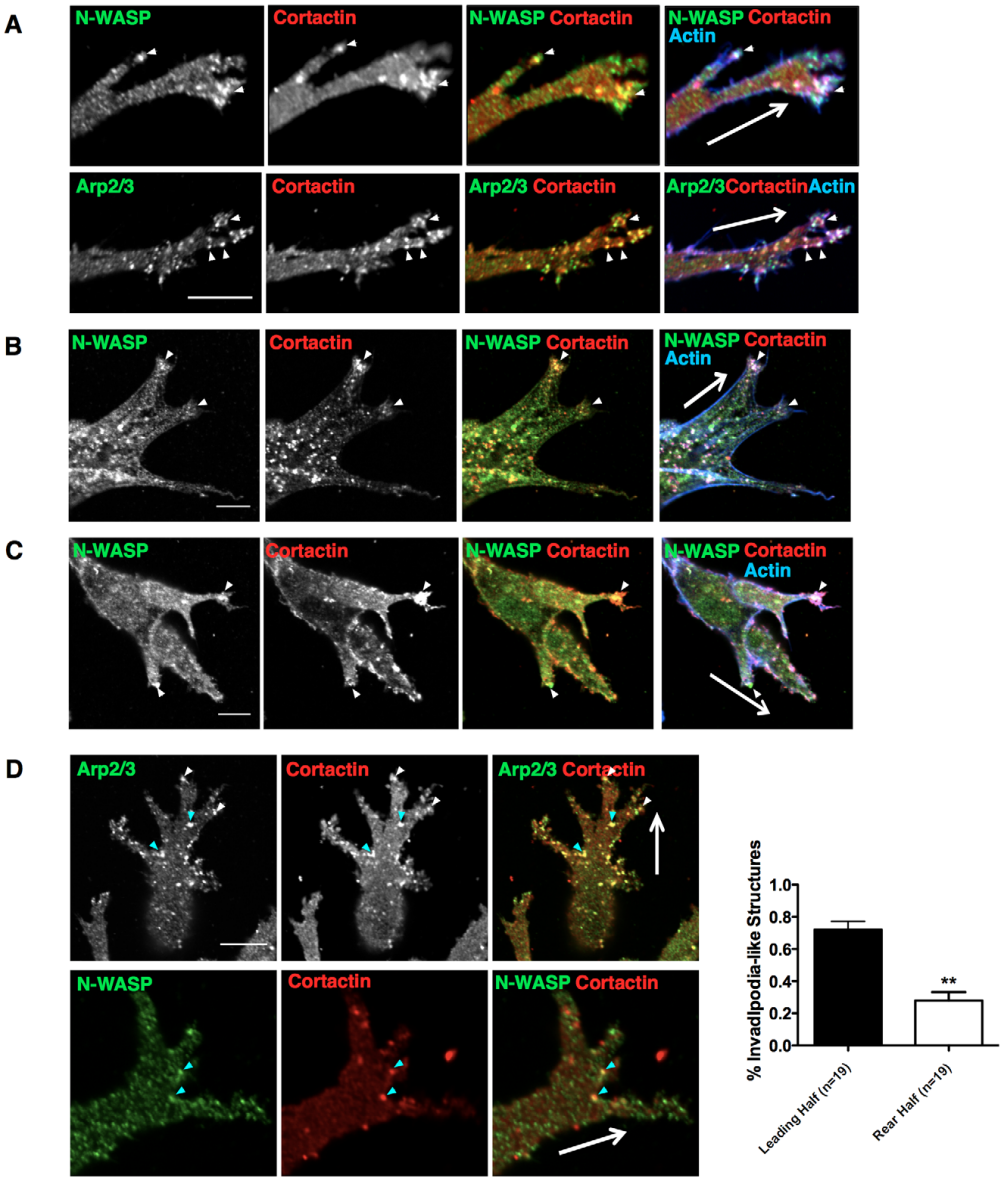
Actin-rich puncta containing cortactin, Arp2/3 complex and N-WASP are apparent in CIA. (A–C) Cells invading under Matrigel in CIA are fixed and stained with proteins previously localized to invadopodia, cortactin (red), N-WASP (green), Arp2/3 component p34-Arc (green) and actin (blue) in a number of cell lines including MDA-MB-231 (A), CHL1 (B), HT1080 (C). (D) Invadopodia in CIA localize at the front of the invading pseudopods (white arrowhead) and branching sites of the pseudopods (blue arrowhead). Quantification shows there are more invadopodia in the front half of the cells than the rear half. Cells were analyzed in three independent experiments. All error bars indicate means ± SD; **, P<0.01 by Student's t-test. All scale bars 20 µm.

Invadopodia are cytoskeletal structures enriched in filamentous actin and multiple actin-associated proteins. We probed for a subset of established components of invadopodia: N-WASP, cortactin and Arp2/3 complex and discovered that they all localized to puncta within invasive pseudopods. These puncta also contained filamentous actin and were often associated with actin spikes (white arrowheads, Fig. 4A; big white arrow indicating the wound direction). These structures weren't cell type specific, as they were present in a range of other cancer cell lines, including CHL-1 melanoma and HT1080 fibrosarcoma cells (Fig. 4B and C). Nearly 100% of these structures formed in contact with the matrix, as opposed to at the interface with the glass, although often they formed near the edges of the cell where it was touching both the glass and the matrix, they almost never formed underneath the cells where they were mostly touching the glass. Quantification in Fig. 4E shows that around 70% of invadopodia in CIA localize to the leading half of the cells (defined as the half facing toward the empty space in the CIA) while only 30% were present at the rear half. We also observed invadopodia-like structures at the branching sites of pseudopods (Fig. 4D). Previous studies show that branches or forks often form where cells encounter physical matrix barriers that require degradation activities [26]. Thus we observe actin-rich puncta and spikes forming at the invasive fronts of cells in CIA that contain proteins also concentrated in invadopodia.

**Figure 5:**
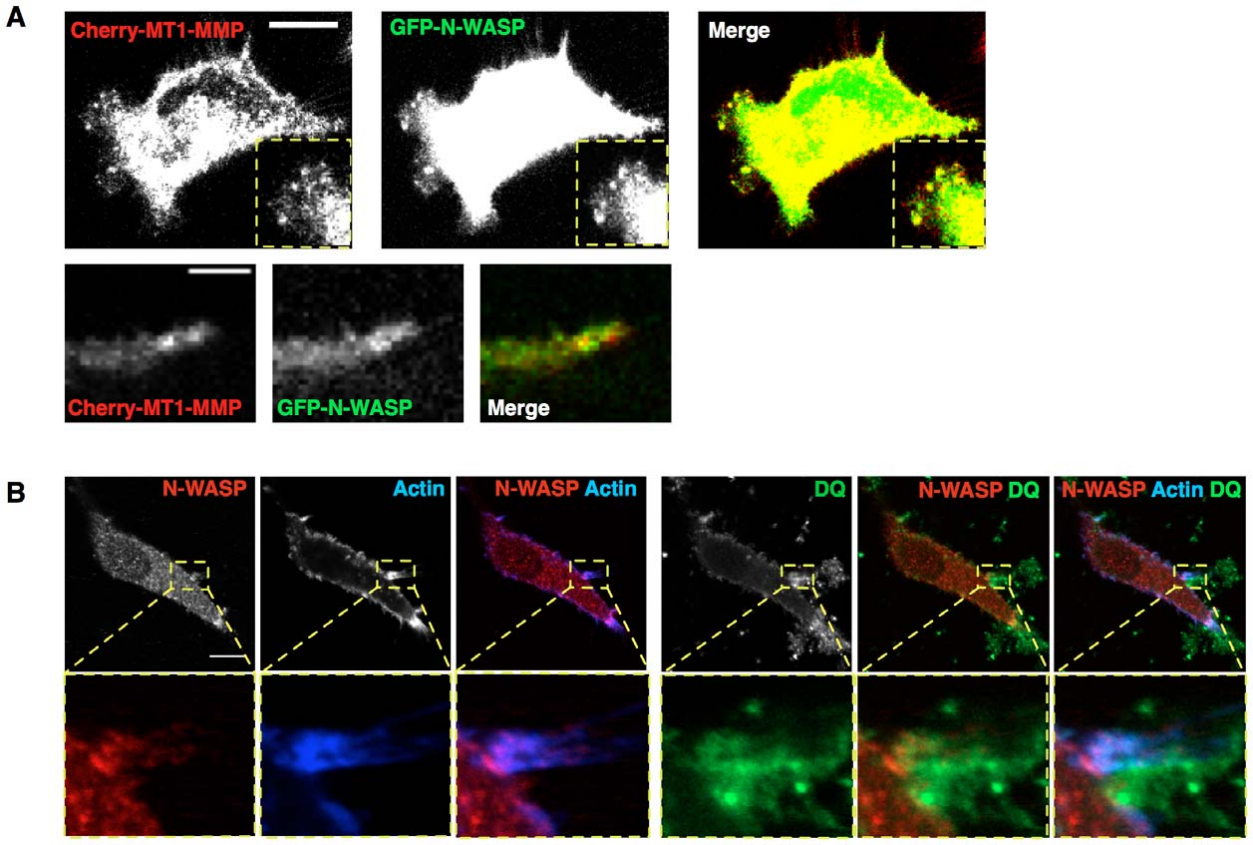
Invadopodia structures in CIA have degradation ability. MDA-MB-231 cells in the CIA assay, showing (A) Cherry-MT1-MMP (red) containing vesicles are delivered to invadopodia structures marked by GFP-N-WASP (green) in CIA. Scale bar 20 µm in the upper panel and 10 µm in the lower panel. (B) DQ collagen is mixed with Matrigel and overlaid on top of the cells in CIA. DQ collagen fluorescence (green) is visualized around some of the actin-rich puncta (blue) with co-localization of N-WASP (red), indicating that they are invadopodia structures in CIA. Scale bar 20 µm.

**Figure 6:**
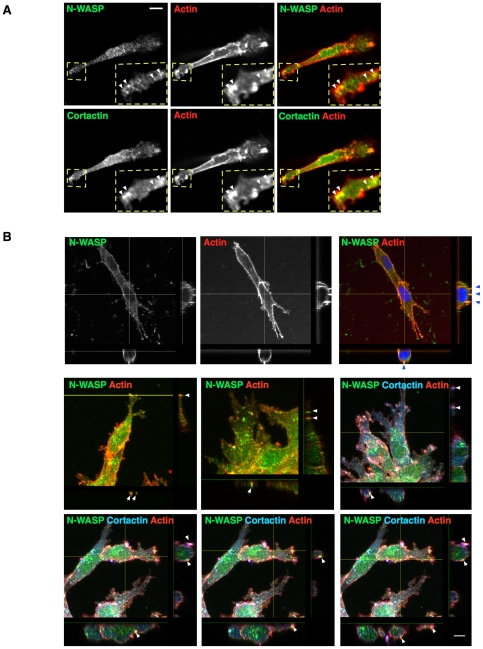
Invadopodia protrude into the matrix in CIA. (A) MDA-MB-231 cells completely embedded in collagen I also show similar elongated morphology as in CIA. Staining of N-WASP or cortactin (green) and actin (red) show that N-WASP and filamentous actin containing structures resembling invadopodia (white arrowheads) are also present in cells migrating in a pure 3D environment. Scale bar 20 µm. (B) Z-stack projection showing invadopodia-like structures (arrowheads) localize to various positions, including the front of the invading pseudopods facing upward into the Matrigel and at the periphery and dorsal surface. Staining shows N-WASP (green), actin (red) and DAPI (blue) or cortactin (blue). See also Movie S8.

One of the important features of invadopodia is that they have matrix-degrading capability [7], [8]. To test whether the puncta observed in CIA were likely to be bona-fide invadopodia, we tested for protease localization with fluorescently tagged MT1-MMP and for protease activity using DQ collagen I, a quenched fluorescent collagen that becomes brighter upon cleavage. mCherry-MT1-MMP puncta co-localized with GFP-N-WASP near the edges of pseudopods facing into the empty space being invaded (Fig. 5A). We also observed DQ collagen fluorescence in the pericellular space, which was concentrated largely near bright F-actin containing protrusions (Fig. 5B). These F-actin protrusions with collagen degradation activity frequently contained multiple invadopodia-like puncta containing N-WASP and Arp2/3 complex, suggesting focal degradation activity does happen at these sites. We cannot rule out that some of the small puncta may be vesicles in transit to sites of matrix degradation, as cortactin, N-WASP and Arp2/3 complex can also localize to intracellular vesicles, as does MT1-MMP [27], [28], [29], [30]. Similar dot-like structures with co-localization of N-WASP, cortactin and actin are also observed in cells embedded in pure 3D collagen or Matrigel environment (Fig. 6A, white arrowheads).

Many of the invadopodia-like puncta locate at the cell periphery and in bright F-actin protrusions, indicating that they interface with the matrix. A 3D reconstruction of cells invading in CIA shows that invading cells typically display a wedge shape with the widest part near the nucleus and with long thin pseudopods, which extend between the glass bottom and the Matrigel (Movie S8). Unlike invadopoda on a 2D gelatin surface, which insert downward into gelatin layer [7], the invadopodia in CIA often extended upward into the overlaid Matrigel (Fig. 6B and Movie S8). Many invadopodia-like structures were observed on the dorsal or side of main cell body, apparently protruding into the Matrigel (Fig. 6B, arrowheads and Movie S8). This is consistent with a previous report that cortactin was more concentrated on the dorsal cell side of cells in CIA [19].

## Discussion

### CIA invasion under Matrigel is a useful assay for the study of cells assuming largely 3D shapes and migration characteristics

We present a straightforward, accessible and quantifiable invasion assay for use with cancer cells in vitro, which is a modification of the CIA [19]. We have further characterized the ability of breast cancer and melanoma cells to make invasive pseudopods, focal adhesions and invadopodia in this assay and the protease dependence of invasion into Matrigel. We find that cells in CIA assume a similar morphology to cells in 3D Matrigel or collagen invasion assays, but that they are easier to image and finer details can be observed due to the glass surface on one side. In general, cells remain associated with the glass during the course of the assay, but they also form extensive interactions with the ECM, including making tunnels and protruding focal adhesions and invadopodia structures into the Matrigel.

Cells in CIA can be observed live in time lapse using fluorescent probes for proteins (such as GFP and mCherry, Fig. 5A) or fixed and stained using antibody probes. Our group has found that this is a considerable advantage over most 3D embedded models, where antibody accessibility can be an issue and staining often appears weak, blurry and lacks a specific discernable pattern. Other useful methods exist for staining cells with antibodies in more true 3D conditions [31], where mammary spheroids are grown in Matrigel. But while these methods may be fine for staining some proteins, such as E-cadherin, they do not work as well in our hands for visualization of the cytoskeleton of migrating cells. As described in Materials and Methods, staining of cells in CIA is straightforward and comparable with standard immunofluorescence staining. Incubation of primary antibody for 3 hours at room temperature or 16 hours at 4°C generally gives consistent and clear staining. Similar to a classical wound healing assay, CIA is directional and quantifiable and it is straightforward to identify the leading front of migrating cells in live or fixed specimens. This is a clear advantage over many methods where cells are embedded in gels and it is difficult to image deep enough into the gel to see the leading cells and to gain a high resolution picture of which direction they are migrating in and of subcellular organelles and structures. In the CIA, the decrease in area of the empty space can be quantified over time, as can the speed of individual cells, allowing quantitative analysis.

#### Cytoskeletal morphology and focal adhesions of cells invading in CIA resemble those of cells invading in 3D

MDA-MB-231 breast cancer cells invaded collectively into the CIA, forming chains of cells that often exceeded 4–5 cells in a row and appeared very similar to cells invading into thick Matrigel plugs in the inverted invasion assay of Hennigan [2]. It seems likely that some cell types invade collectively both in CIA and inverted invasion assays because of the micro-tunnels that are generated, which allow migration into spaces left behind by the leading cells. These cells had small focal adhesions of which approximately 60% associated with the glass interface, often in extending leading pseudopodia. Approximately 40% of the focal adhesions were deeper into the Matrigel and not touching the glass, indicating that cells made significant attachments to the Matrigel and were responding to a 3D matrix. Cells in CIA generally lacked lamellipodia and closely resembled the elongated protrusive phenotype assumed by mesenchymal type cells in 3D.

#### Invasion into Matrigel in CIA is protease dependent and cells form invadopod-like puncta

Migration in the CIA was dependent on MMP activity and in particular, it was partially dependent on MT1-MMP, a transmembrane metalloprotease. Treatment with GM6001 caused around a 50% decrease in cell speed and a significant decrease in the area covered by cells in 30–40 hours of invasion. Depletion of MT1-MMP by siRNA was somewhat less effective, with around a 30% decrease in speed and a smaller but significant decrease in area covered. The reasons for a partial rather than total dependence on protease for migration in CIA are likely multiple. Firstly, we used Matrigel for this assay and MT1-MMP is a collagenase, while the complex mixture of Matrigel also contains other components such as laminin (50–60%), collagen IV (30%), entactin (8%) (BD Bioscience website and [32]). Secondly, the cells are partially adherent to a glass surface, so they might use squeezing forces (between the gel and the glass) to move as well as remodeling forces. Thirdly, cells might be able to squeeze between spaces in the gel, just as they do in 3D, although Matrigel in general has very small spaces, but the fibers in Matrigel are not covalently crosslinked as they would be in real basement membranes in vivo [9].

Conversely, there could be multiple reasons why cells need metalloproteases to crawl in CIA, including degradation of a physical matrix barrier [33], [34], [35], [36], cleavage of cell surface receptors that lead to enhancement of motility across matrix [36], [37] and cleavage of matrix components that release pro-motility factors [38]. We observed both melanoma and breast cancer cells forming putative invadopodia structures in this assay, which were rich in N-WASP, cortactin, Arp2/3 complex and actin. These structures formed throughout cells, but were most prominent in the half of the cell facing into the direction of overall migration. They were commonly found near tips of pseudopodia and at places where pseudopods forked. Our results support the idea, but do not prove, that these are bona fide invadopodia, as we could visualize N-WASP puncta co-localizing with MT1-MMP in leading pseudopodia and quenched fluorescent collagen revealed degradation of matrix taking place in the pseudopodia near N-WASP and actin-rich puncta. We rarely if ever observed invadopodia structures residing at the glass interface, but rather they always protruded into the Matrigel. We also observed similar structures in 3D collagen gels and so have other groups [17], [18], [14] suggesting that these represent cytoskeletal foci involved in matrix degradation and protrusion and represent invadopodia in 3D.

A previous study has shown that macrophages also assume a mesenchymal mode while invading in ECM [39]. Concomitantly, these cells form a number of elongated protrusions with F-actin enriched dot-like structures at their tips with co-localizations of typical podosome components. This is strikingly similar to what we observed with MDA-MB-231 cells suggesting cells invade in CIA in a fashion similar to 3D. MDA-MB-231 cells have been observed to make finger-like protrusions when they invade in Matrigel [40], [41], however whether matrix degradation takes places at the bases of these protrusions and recruitment of matrix-lytic proteases has not been demonstrated. Taking advantage of CIA, we have clearly shown that many invadopodia proteins localized to the dot-like invadopodia structures. With quenched fluorescent collagen, we have demonstrated that these dot-shape structures reside next to matrix lysis activity. Furthermore, we have shown that vesicles containing major pericellular collagenase MT1-MMP localizing in the proximity of invadopodia (Fig. 5A). This observation agrees with previous study by Bravo-Cordero where they observe vesicles with MT1-MMP are recruited to the contact site of cell membrane with collagen meshwork [27].

In summary, we have made some minor modifications to the CIA assay [19] that we hope will make it useful to groups for quantification and visualization of the invasion process. We have also obtained high quality images and demonstrated that CIA closely mimics a 3D environment for cells to invade and it is an excellent platform to study individual cell invasion behavior, which can be more difficult with other invasion assays. This assay provides a 3D context but includes straightforward execution and direct imaging, which we believe will greatly help to study cancer cell invasion in detail and improve our understanding of this important process.

## Supporting Information

Movie S1 **MDA-MB-231 cells invading in CIA.** Cells display elongated morphology and form finger-like collective migration chains. Images were acquired every 3 min for 18 hours. Elapsed time is indicated.
(MOV)

Movie S2 **Single MDA-MB-231 cells actively remodeling Matrigel in CIA.** Enlarged area shows single cells extending long pseudopods to re-shape Matrigel. Micro-tunnels are clearly seen as a result of cells remodeling the matrix. Images were acquired every 3 min for 17 hours and 18 minutes. Elapsed time is indicated.
(MOV)

Movie S3 **3D reconstruction movie of MDA-MB-231 cells invading in a Matrigel plug of inverted invasion assay.** Cells display elongated cylinder-like morphology and form cell invasion chains, similar to cells in CIA.
(MOV)

Movie S4 **MDA-MB-231 cells migrate randomly on a 2D rigid surface coated with fibronectin.** Cells appear flat and spread with fan-like lamellipodia. Images were acquired every 3 min for approximately 7 hours.
(MOV)

Movie S5 **3D reconstruction of FA staining of cells on a 2D surface.** MDA-MB-231 cells are labeled with focal adhesion (FA) marker phospho-paxillin (red), actin (green) and DNA (blue). Movie shows FA displaying different organization and localization in cells migrating on 2D surface shown here compared to cells invading in CIA (shown in Movie S6).
(MOV)

Movie S6 **3D reconstruction of FA staining of cells in CIA.** MDA-MB-231 cells are labeled with focal adhesion (FA) marker phospho-paxillin (red), actin (green) and DNA (blue). Movie shows FA displaying different organization and localization in cells migrating on 2D surface (shown in Movie S5) compared with cells invading in CIA (shown here).
(MOV)

Movie S7 **Cells invade with or without metalloprotase inhibitor GM6001 in CIA.** Addition of GM6001 at concentrations of 25 µM greatly retards MDA-MB-231 cell invasion in the period of 37 hours (GM6001 right hand side, controls left side). Images were acquired every 3 min. Elapsed time is indicated.
(MOV)

Movie S8 **3D reconstruction movie shows invadopodia-like structures in MDA-MB-231 cells invading in CIA.** These cells are labeled with actin (red) and N-WASP (green) to view the invadopodia-like structures. Co-localization of actin puncta and N-WASP (yellow) represents invadopodia-like actin protrusions, which are observed on the invasive pseudopods, dorsal face and side.
(MOV)

## References

[1] Kleinman HK, Jacob K (2001). Invasion assays..

[2] Hennigan RF, Hawker KL, Ozanne BW (1994). Fos-transformation activates genes associated with invasion.. Oncogene.

[3] Caswell PT, Spence HJ, Parsons M, White DP, Clark K (2007). Rab25 associates with alpha5beta1 integrin to promote invasive migration in 3D microenvironments.. Developmental cell.

[4] Li A, Dawson JC, Forero-Vargas M, Spence HJ, Yu X (2010). The actin-bundling protein fascin stabilizes actin in invadopodia and potentiates protrusive invasion.. Current biology: CB.

[5] Buccione R, Caldieri G, Ayala I (2009). Invadopodia: specialized tumor cell structures for the focal degradation of the extracellular matrix.. Cancer metastasis reviews.

[6] Caldieri G, Ayala I, Attanasio F, Buccione R (2009). Cell and molecular biology of invadopodia.. International review of cell and molecular biology.

[7] Linder S (2007). The matrix corroded: podosomes and invadopodia in extracellular matrix degradation.. Trends in cell biology.

[8] Weaver AM (2006). Invadopodia: specialized cell structures for cancer invasion.. Clinical & experimental metastasis.

[9] Wolf K, Friedl P (2009). Mapping proteolytic cancer cell-extracellular matrix interfaces.. Clin Exp Metastasis.

[10] Wolf K, Wu YI, Liu Y, Geiger J, Tam E (2007). Multi-step pericellular proteolysis controls the transition from individual to collective cancer cell invasion.. Nat Cell Biol.

[11] Fisher KE, Sacharidou A, Stratman AN, Mayo AM, Fisher SB (2009). MT1-MMP- and Cdc42-dependent signaling co-regulate cell invasion and tunnel formation in 3D collagen matrices.. J Cell Sci.

[12] Gaggioli C, Hooper S, Hidalgo-Carcedo C, Grosse R, Marshall JF (2007). Fibroblast-led collective invasion of carcinoma cells with differing roles for RhoGTPases in leading and following cells.. Nature cell biology.

[13] Friedl P, Wolf K (2003). Tumour-cell invasion and migration: diversity and escape mechanisms.. Nature reviews Cancer.

[14] Burridge K, Chrzanowska-Wodnicka M (1996). Focal adhesions, contractility, and signaling.. Annual review of cell and developmental biology.

[15] Bershadsky AD, Ballestrem C, Carramusa L, Zilberman Y, Gilquin B (2006). Assembly and mechanosensory function of focal adhesions: experiments and models.. European journal of cell biology.

[16] Parsons JT, Horwitz AR, Schwartz MA (2010). Cell adhesion: integrating cytoskeletal dynamics and cellular tension.. Nature reviews Molecular cell biology.

[17] Fraley SI, Feng Y, Krishnamurthy R, Kim DH, Celedon A (2010). A distinctive role for focal adhesion proteins in three-dimensional cell motility.. Nature cell biology.

[18] Kubow KE, Horwitz AR (2011). Reducing background fluorescence reveals adhesions in 3D matrices.. Nat Cell Biol.

[19] Kam Y, Guess C, Estrada L, Weidow B, Quaranta V (2008). A novel circular invasion assay mimics in vivo invasive behavior of cancer cell lines and distinguishes single-cell motility in vitro.. BMC cancer.

[20] Fisher KE, Sacharidou A, Stratman AN, Mayo AM, Fisher SB (2009). MT1-MMP- and Cdc42-dependent signaling co-regulate cell invasion and tunnel formation in 3D collagen matrices.. Journal of cell science.

[21] Scott RW, Hooper S, Crighton D, Li A, Konig I (2010). LIM kinases are required for invasive path generation by tumor and tumor-associated stromal cells.. J Cell Biol.

[22] Buccione R, Orth JD, McNiven MA (2004). Foot and mouth: podosomes, invadopodia and circular dorsal ruffles.. Nat Rev Mol Cell Biol.

[23] Chen WT (1989). Proteolytic activity of specialized surface protrusions formed at rosette contact sites of transformed cells.. J Exp Zool.

[24] Linder S (2007). The matrix corroded: podosomes and invadopodia in extracellular matrix degradation.. Trends Cell Biol.

[25] Clark ES, Weaver AM (2008). A new role for cortactin in invadopodia: regulation of protease secretion.. Eur J Cell Biol.

[26] Friedl P, Wolf K (2009). Proteolytic interstitial cell migration: a five-step process.. Cancer metastasis reviews.

[27] Bravo-Cordero JJ, Marrero-Diaz R, Megias D, Genis L, Garcia-Grande A (2007). MT1-MMP proinvasive activity is regulated by a novel Rab8-dependent exocytic pathway.. The EMBO journal.

[28] Benesch S, Lommel S, Steffen A, Stradal TE, Scaplehorn N (2002). Phosphatidylinositol 4,5-biphosphate (PIP2)-induced vesicle movement depends on N-WASP and involves Nck, WIP, and Grb2.. The Journal of biological chemistry.

[29] Benesch S, Polo S, Lai FP, Anderson KI, Stradal TE (2005). N-WASP deficiency impairs EGF internalization and actin assembly at clathrin-coated pits.. Journal of cell science.

[30] Taunton J, Rowning BA, Coughlin ML, Wu M, Moon RT (2000). Actin-dependent propulsion of endosomes and lysosomes by recruitment of N-WASP.. The Journal of cell biology.

[31] Debnath J, Muthuswamy SK, Brugge JS (2003). Morphogenesis and oncogenesis of MCF-10A mammary epithelial acini grown in three-dimensional basement membrane cultures.. Methods.

[32] Kleinman HK, McGarvey ML, Liotta LA, Robey PG, Tryggvason K (1982). Isolation and characterization of type IV procollagen, laminin, and heparan sulfate proteoglycan from the EHS sarcoma.. Biochemistry.

[33] Sabeh F, Ota I, Holmbeck K, Birkedal-Hansen H, Soloway P (2004). Tumor cell traffic through the extracellular matrix is controlled by the membrane-anchored collagenase MT1-MMP.. The Journal of cell biology.

[34] Hotary K, Li XY, Allen E, Stevens SL, Weiss SJ (2006). A cancer cell metalloprotease triad regulates the basement membrane transmigration program.. Genes & development.

[35] Ueda J, Kajita M, Suenaga N, Fujii K, Seiki M (2003). Sequence-specific silencing of MT1-MMP expression suppresses tumor cell migration and invasion: importance of MT1-MMP as a therapeutic target for invasive tumors.. Oncogene.

[36] Zaman MH, Trapani LM, Sieminski AL, Mackellar D, Gong H (2006). Migration of tumor cells in 3D matrices is governed by matrix stiffness along with cell-matrix adhesion and proteolysis.. Proceedings of the National Academy of Sciences of the United States of America.

[37] Marrero-Diaz R, Bravo-Cordero JJ, Megias D, Garcia MA, Bartolome RA (2009). Polarized MT1-MMP-CD44 interaction and CD44 cleavage during cell retraction reveal an essential role for MT1-MMP in CD44-mediated invasion.. Cell motility and the cytoskeleton.

[38] Barbolina MV, Stack MS (2008). Membrane type 1-matrix metalloproteinase: substrate diversity in pericellular proteolysis.. Semin Cell Dev Biol.

[39] Van Goethem E, Poincloux R, Gauffre F, Maridonneau-Parini I, Le Cabec V (2010). Matrix architecture dictates three-dimensional migration modes of human macrophages: differential involvement of proteases and podosome-like structures.. Journal of immunology.

[40] Lizarraga F, Poincloux R, Romao M, Montagnac G, Le Dez G (2009). Diaphanous-related formins are required for invadopodia formation and invasion of breast tumor cells.. Cancer research.

[41] Poincloux R, Lizarraga F, Chavrier P (2009). Matrix invasion by tumour cells: a focus on MT1-MMP trafficking to invadopodia.. Journal of cell science.

